# Variable and maximum blood glucose levels during the first week after ICU admission are related to the severity of the patients

**DOI:** 10.1186/cc10778

**Published:** 2012-03-20

**Authors:** M Hoshino, Y Haraguchi, K Oda, S Kajiwara

**Affiliations:** 1 Shisei Hospital, Saitama, Japan; 2 Geriatric Health Services Facility, Kokubunnji-shi, Japan

## Introduction

Significance of blood glucose (BG) control in ICU patients, especially in terms of mortality reduction, has been recognized in recent years. However, the relationship between BG profile and the severity, as well as BG target, is not clearly elucidated. A preliminary study was performed in order to clarify the relationship between BG profile and the severity of ICU patients.

## Methods

Forty-seven patients were studied. The following parameters were calculated during first week after ICU admission. (1) Mean and maximum value of SOFA scores (SOFAm and SOFAmax, respectively). (2) Mean, standard deviation, maximum, minimum, and difference of BG levels (BGm, BGsd, BGmax, BGmin, BGd(BGmax - BGmin), respectively). BG levels were measured basically every 6 hours with capillary blood. (3) Correlation coefficients (*r*) between the abovementioned SOFA scores and BG parameters were calculated using two-dimensional and linear regression analysis (*r*_t_, *r*_l_, respectively).

## Results

**(**1) *r*_t _and *r*_l _(*r*_t_/*r*_l_) between SOFAmax and BG parameters: BGsd (0.48/0.36), BGmax (0.47/0.33), BGd (0.47/0.35), BGm (0.30/0.22), BGmin (0.21/0.36). (2) (*r*_t_/*r*_l_) between SOFAm and BG parameters: BGsd (0.45/0.33), BGmax (0.45/0.28), BGd (0.45/0.30), BGm (0.28/0.17), BGmin (0.17/0.29).

## Conclusion

**(**1) Variable and maximum BG levels during the first week (BGsd, BGd, BGmax), rather than mean and minimum BG levels (BGm, BGmin), were related to the severity. (2) Suppression of the higher variable and maximum BG levels was considered to link to better outcome. (3) On the other hand, part of the severe patients seemed to have the lowest of those variable and maximum BG levels, judging from two-dimensional or parabolic correlations between those BG parameters and SOFA scores.

